# Complete Genome Sequence of the Hyperthermophilic Sulfate-Reducing Bacterium *Thermodesulfobacterium geofontis* OPF15^T^

**DOI:** 10.1128/genomeA.00162-13

**Published:** 2013-04-11

**Authors:** James G. Elkins, Scott D. Hamilton-Brehm, Susan Lucas, James Han, Alla Lapidus, Jan-Fang Cheng, Lynne A. Goodwin, Sam Pitluck, Lin Peters, Natalia Mikhailova, Karen W. Davenport, John C. Detter, Cliff S. Han, Roxanne Tapia, Miriam L. Land, Loren Hauser, Nikos C. Kyrpides, Natalia N. Ivanova, Ioanna Pagani, David Bruce, Tanja Woyke, Robert W. Cottingham

**Affiliations:** BioEnergy Science Center, Oak Ridge National Laboratory, Oak Ridge, Tennessee, USAa;; Biosciences Division, Oak Ridge National Laboratory, Oak Ridge, Tennessee, USAb;; U.S. Department of Energy Joint Genome Institute, Walnut Creek, California, USAc;; Bioscience Division, Los Alamos National Laboratory, Los Alamos, New Mexico, USAd

## Abstract

*Thermodesulfobacterium geofontis* OPF15^T^ (ATCC BAA-2454, JCM 18567) was isolated from Obsidian Pool, Yellowstone National Park, and grows optimally at 83°C. The 1.6-Mb genome sequence was finished at the Joint Genome Institute and has been deposited for future genomic studies pertaining to microbial processes and nutrient cycles in high-temperature environments.

## GENOME ANNOUNCEMENT

Sulfate-reducing microorganisms (SRM) are ubiquitous in anoxic environments and play a key role not only in the sulfur cycle, but also in driving the decomposition of organic matter through trophic interactions (1). Thermal environments, including terrestrial hot springs, also support SRM from the bacterial and archaeal domains. Currently, five type strains have been described within the genus *Thermodesulfobacterium*: *Thermodesulfobacterium commune* YSRA-1, *Thermodesulfobacterium hveragerdense* JSP, *Thermodesulfobacterium hydrogeniphilum* SL6, *Thermodesulfobacterium thermophilum* DSM1276 (2), and the recently described *Thermodesulfobacterium geofontis* OPF15 (3). Previously, no complete genome sequences from organisms within the genus *Thermodesulfobacterium* have been released. *Thermodesulfatator indicus* CIR29812^T^ has the most closely related genome representing a thermophilic sulfate-reducing bacterium (4).

Isolation attempts from Obsidian Pool enrichment cultures (85°C) produced a strain with 99.7% 16S rRNA gene sequence identity to the environmental clone OPB45 (accession no. AF027096.1), originally deposited by Hugenholtz et al. (5). The isolate utilizes hydrogen or formate as an electron donor while it reduces sulfate, thiosulfate, or elemental sulfur to sulfide. Carbon dioxide is required for its growth, while organic acids and alcohols are not used as electron donors.

The draft genome sequence of *T. geofontis* OPF15^T^ (originally designated *Thermodesulfobacterium* sp. OPB45) was generated at the U.S. Department of Energy (DOE) Joint Genome Institute (JGI) using a combination of Illumina (6) and 454 DNA sequencing technologies (7). For this genome, we constructed an Illumina GA II shotgun library that generated 80,058,940 reads and totaled 6,084.5 Mb, a 454 Titanium library that generated 272,891 reads, and 1 paired-end 454 library with an average insert size of 7 kb that generated 297,746 reads and totaled 140.6 Mb of 454 data. The initial draft assembly contained 17 contigs in 1 scaffold. The 454 Titanium standard data and the 454 paired-end data were assembled together with Newbler v2.3-PreRelease-6/30/2009. The Newbler consensus sequences were computationally shredded into 2-kb overlapping fake reads (shreds). Illumina sequencing data were assembled with Velvet v1.0.13 (8), and the consensus sequences were computationally shredded into 1.5-kb overlapping fake reads (shreds). We integrated the 454 Newbler consensus shreds, the Illumina Velvet consensus shreds, and the read pairs in the 454 paired-end library using parallel Phrap vSPS 4.24 (High Performance Software, LLC). The software Consed (9–11) was used in the finishing process, as described previously (12). A total of 75 additional PCRs were necessary to close all gaps. The final assembly is based on 59.8 Mb of 454 draft data, which provides an average of 37.4× coverage of the genome, and 5,985.5 Mb of Illumina draft data, which provides an average of 3,740.9× coverage.

The circular contiguous chromosome contains 1,634,377 bp and a G+C content of 30.59%. No extrachromosomal elements were discovered. The genome was annotated using Prodigal at Oak Ridge National Laboratory (13), which identified 1,635 candidate protein-encoding gene models. Further analysis of the genome should give new insights into the ecophysiology and genomics of deep-branching SRM and their role in high-temperature environments.

### Nucleotide sequence accession number. 

The final annotated genome sequence of *T. geofontis* OPF15^T^ has been deposited in GenBank under the accession no. CP002829.
